# Archaeal Assemblages Inhabiting Temperate Mixed Forest Soil Fluctuate in Taxon Composition and Spatial Distribution over Time

**DOI:** 10.1155/2013/870825

**Published:** 2013-08-01

**Authors:** Colby A. Swanson, Marek K. Sliwinski

**Affiliations:** Biology Department, University of Northern Iowa, MSH 17, Cedar Falls, IA 50614, USA

## Abstract

This study explored the persistence and spatial distribution of a diverse Archaeal assemblage inhabiting a temperate mixed forest ecosystem. Persistence under native conditions was measured from 2001 to 2010, 2011, and 2012 by comparison of 16S rRNA gene clone libraries. The Archaeal assemblages at each of these time points were found to be significantly different (AMOVA, *P* < 0.01), and the nature of this difference was dependent on taxonomic rank. For example, the cosmopolitan genus g_*Ca.* Nitrososphaera (I.1b) was detected at all time points, but within this taxon the abundance of s_SCA1145, s_SCA1170, and s_*Ca.* N. gargensis fluctuated over time. In addition, spatial heterogeneity (patchiness) was measured at these time points using 1D TRFLP-SSCP fingerprinting to screen soil samples covering multiple spatial scales. This included soil collected from small volumes of 3 cubic centimeters to larger scales—over a surface area of 50 m^2^, plots located 1.3 km apart, and a separate locality 23 km away. The spatial distribution of Archaea in these samples changed over time, and while g_*Ca.* Nitrososphaera (I.1b) was dominant over larger scales, patches were found at smaller scales that were dominated by other taxa. This study measured the degree of change for Archaeal taxon composition and patchiness over time in temperate mixed forest soil.

## 1. Introduction

Our understanding of Archaea inhabiting soils has expanded exponentially in the last few decades through the use of culture-independent molecular tools. This has led to the discovery of novel Archaeal lineages in terrestrial environments [1] including the recently recognized phylum, Thaumarchaeota [2, 3], members of which have subsequently been shown to play a vital role in the global nitrogen cycle by performing the rate-limiting step for nitrification in most soils [4]. Within the last decade Archaeal phylogeny has been greatly improved by the successful cultivation of Thaumarchaeota in isolation and as dominant members of enrichments from a number of environments. These environments vary, for example, marine, *Nitrosopumilus maritimus* [5]; hot springs in North America and Siberia, *Nitrosocaldus yellowstonii *and *Nitrososphaera gargensis* [6, 7]; and recently from a number of different mesophilic soil sites, *Ca*. Nitrosoarchaeum koreensis, *Ca. *Nitrosotalea devanaterra, and *Ca.* Nitrososphaera viennensis strain EN76 and JG1 [8–11]. In terms of cultivated species representing the most abundant soil taxa, g_*Ca.* Nitrososphaera (I.1b), s_*Ca.* N. gargensis has three cultured representatives [7, 8, 11], s_SCA1145 was enriched but at low relative abundance [12], and s_SCA1170 has not yet been cultivated in a laboratory setting. The second dominant soil clade, o_NRP-J (I.1c), is genetically more diverse than g_*Ca.* Nitrososphaera (I.1b) but does not yet contain a cultivated representative. The success in cultivating Thaumarchaeota species has allowed for genomic studies, most recently the genome of *Nitrososphaera gargensis* has been reported and compared to the other Thaumarchaeota genomes available [13]. In total, these studies have led to unprecedented insight into the evolution and physiology of g_*Ca.* Nitrososphaera (I.1b), but the ecological significance of other Archaeal lineages in soils is yet to be determined. In this study, we explored the persistence and spatial distribution of soil Archaea to determine whether taxon composition and patchiness is stable. By better defining niche segregation for different Archaeal lineages over time and space, future studies can target the autecology of these lineages in soil.

While we are not aware of other studies that have directly measured temporal dynamics of soil Archaea at a single locality over multiple years, some temporal information can be gleaned from comparison of the 16S rRNA genes deposited in Genbank. For example, clone SCA1145, a member of g_*Ca.* Nitrososphaera (I.1b), was isolated in 1995 from arable soil collected in WI, USA [14]. This sequence has been subsequently amplified by labs working independently on different continents. Even with a stringent definition for phylotype such as 100% sequence identity over 1300 nucleotides, SCA1145 has been found over multiple years from 1995 to 2010 including Austria, 2005 (RotA-75iia, DQ278116); Mexico, 2009 (TX1C04, FJ784302); and Japan, 2010 (K09_0_56, AB541694). While this shows SCA1145 has been repeatedly sampled at the global level, it is not known whether this pattern results from a persistent population established at each locality or the chance capture of a species in constant flux. 

At the microbial level, the world's soils represent an immensely heterogeneous environment filled with microhabitats that can vary from one sand grain to the next. While this was once thought of as an obstacle to understanding soil ecology, an unwelcome source of variability when sampling microbes in native environments, over time the importance of measuring spatial variability and using the resulting knowledge to guide sampling strategies has become more apparent [15]. In terms of soil, small-scale sampling of ammonium- and nitrite-oxidizing bacteria has shown spatial structure can exist at the millimeter scale [16], while large-scale sampling at the global level indicates the most diverse Archaeal assemblages occur within forests/shrublands in contrast to deserts/dry valleys, agricultural fields, and grasslands/prairies [17]. In this study, we used multiscale sampling of temperate mixed forest sites, known to harbor a diverse assemblage of soil Archaea, to measure persistence over multiple time points. In addition, the temporal spatial distribution of soil Archaea was compared in small volumes of 3 cubic centimeters and larger scales—over a surface area of 50 m^2^, plots located 1.3 km apart, and a separate locality 23 km away.

## 2. Materials and Methods

### 2.1. Sample Collection

**Figure 1:**
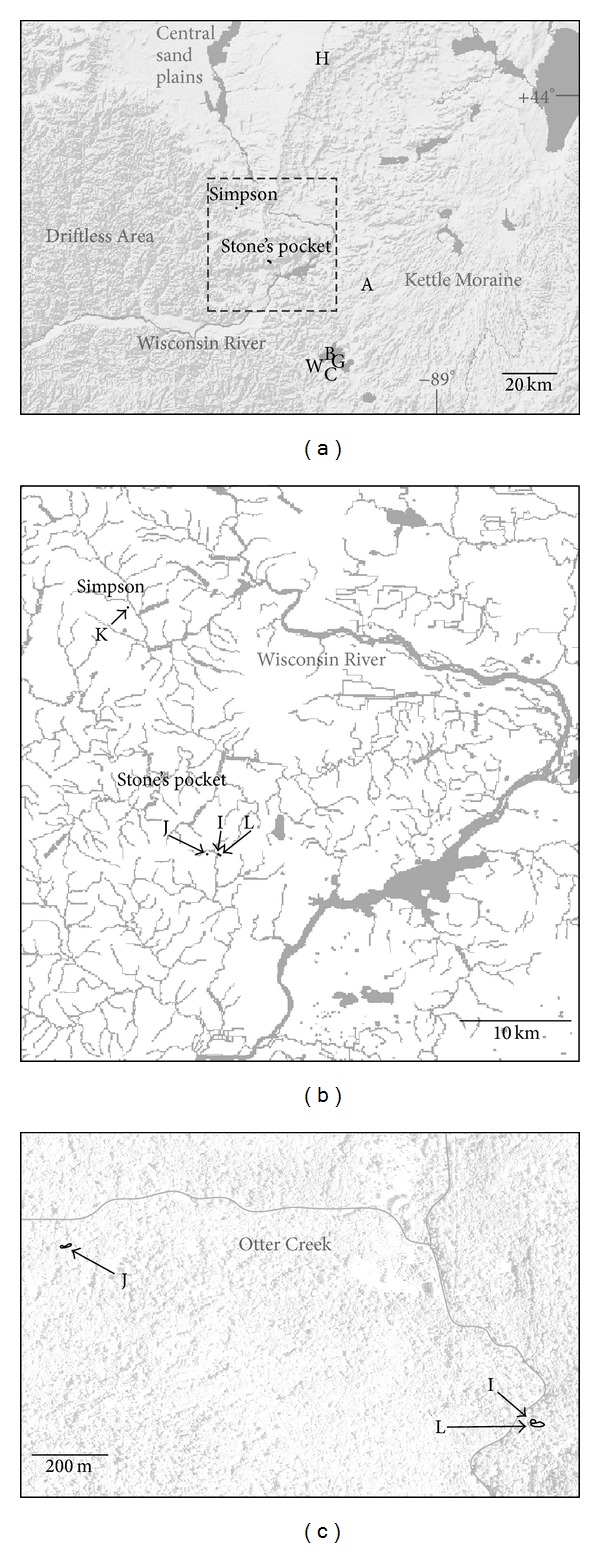
Map indicating the locations of Stone's Pocket and Simpson, the two localities sampled over time in this study. (a) Map of central Wisconsin including the sampling sites labeled A, B, C, G, H, and W that were sampled previously. (b) Close-up of the area outlined in map A. Plots L, I, J, and K were sampled in this study. (c) Close-up of sampling plots at the Stone's Pocket locality.

Collection sites for this study, Stone's Pocket and Simpson, were located near the previously sampled sites: A, B, C, G, H, and W which were tested for Archaea and shown to harbor only g_*Ca.* Nitrososphaera (I.1b) [18]. These sites represent a variety of soils formed by different geological processes (Table 1). Stone's Pocket and Simpson are part of the Driftless Area in central Wisconsin, USA, which was surrounded but not covered by glaciers during the last ice age (Figure 1(a)). The Kettle Moraine area was formed when two glacial lobes collided then receded leaving glacial sediments and pits (kettles) gouged into the earth. Sampling sites A, B, C, G, and W are located on soils at the edge of the Kettle Moraine adjacent to the Driftless Area. The Hancock sampling site (H) is located on the sandy outwash deposited by a proglacial lake which drained in a catastrophic flood along the current path of the Wisconsin river when the glaciers receded ~14,000 years ago.

The Stone's Pocket locality is adjacent to the Baxter's Hollow Nature Conservancy in the Wisconsin Baraboo Foothills and is 43 km away from West Madison Agricultural Research Station (site W in Figure 1(a)) where SCA1145 was initially discovered in 1995 [14], but, unlike the research station, this site has remained under native conditions over the course of this longitudinal study. The Simpson locality is also a mixed temperate forest site but is located in a separate watershed 23 km northwest, adjacent to the Dell Creek State Wildlife Area (Figure 1(b)). Three plots, designated L, I, and J, were sampled at the Stone's Pocket locality (Figure 1(c)). Plots L and I are adjacent to each other, and plot J is 1.3 km away. A fourth plot, designated K, was sampled at the Simpson collection site. In 2001, soil samples were collected throughout the plots I, J, L, and K as part of a separate study [19]. Plot boundaries were defined in the field at this time based on the smallest area that included the following diverse plant lineages: lycopod (club moss), pteridophyte (fern), gymnosperm (conifer), dicotyledonous (dicots), and monocotyledonous (monocots). The surface area of the resulting plots was 10, 35, 50, and 15 m^2^ for plots I, J, L, and K, respectively. Plots were resampled yearly beginning in 2010 at the same time of year, late Fall, during the last week of November, except plot K which was not sampled in 2010. Each year, replicate soil samples were collected along a transect spanning each plot. Some replicates from each plot were used in this study, while the remaining replicates are being stored at −80°C for future studies spanning longer time periods.

Soil samples were collected from the surface layer of soil by first removing overlaying plant debris such as leaves and twigs. Each soil sample was collected using autoclaved supplies from an area ~1.5 cm wide and ~1.5 cm deep by mixing the soil with a metal microspatula to homogenize ~3 cubic centimeters (cm^3^) of soil. This homogenized soil was then transferred into a microcentrifuge tube and flash frozen in the field using either liquid nitrogen (samples collected in 2001) or dry ice (samples collected from 2010 to 2012). Soil samples were stored in the laboratory at −80°C until DNA extraction. 

### 2.2. DNA Amplification and Fingerprinting

DNA was extracted from 100–250 mg of each soil sample using the PowerSoil DNA isolation kit (MO BIO Laboratories) following the manufacturer's protocol. DNA concentration and purity were determined using a NanoVue spectrophotomer and then diluted to 5 ng/*μ*L reaction. DNA template was amplified with Phusion Hot Start II using High-Fidelity buffer. Reactions included 1x HF buffer, 0.2 mM dNTPs, 5 pmol forward primer, 5 pmol reverse primer, 2 mg nonacetylated BSA (Ambion), 5 ng template DNA, and 0.4 U Phusion Hot Start II polymerase (New England BioLabs Inc.) in a final volume of 15 *μ*L. PCR conditions included an initial denaturation step of 98°C 30 s, followed by forty cycles of 98°C 10 s, 55°C 10 s, and 72°C 30 s, and followed by a final extension of 72°C for 5 m. For SSCP, DNA templates were amplified using primers 133F/248R as described previously [18]. For fingerprinting by One-Dimensional Terminal Restriction Fragment Length Polymorphism–Single Stranded Conformation Polymorphism (1D TRFLP-SSCP) and for clone library construction, DNA templates were amplified using primers 133F and 1492R and with the extension time increased to 1 m 20 s. Some samples did not produce PCR products for DNA fingerprinting and were excluded from analysis; these include a 2001 plot I replicate, a 2011 plot I replicate, and a 2012 plot K replicate. 

1D TRFLP-SSCP is a combination of profiling methods which provides a higher dynamic range by generating a SSCP profile for each TRFLP phylotype on a single gel. We tested the utility of this approach for distinguishing the 2001 and 2010 clone libraries *in silico* and determined that a double digest using the enzymes CfoI and ApaI is able to differentiate the greatest number of sequences from broad taxonomic groups. All of the sequences in Figure 4 affiliated with g_*Ca. *Nitrososphaera (I.1b) would produce a single TRFLP phylotype of 206 bp; the o_NRP-J (I.1c) sequences would produce fragments of 90, 180, 200, and 240, while the unclassified Archaea (UA) sequence would produce a fragment of 109 bp.

DNA fingerprinting was conducted following [18]. The forward primer, 133F, included an infrared IRDye-label (IRD700) for detection on a Licor DNA analyzer and six phosphorothioate bonds on the 5′ end to prevent nonspecific digestion by lambda exonuclease. The reverse primers, 248R and 1492R, were 5′ phosphorylated to enable selective lambda exonuclease digestion of the complementary DNA strand. Restriction digests and lambda exonuclease digests (New England Biolabs) were conducted according to manufacturer's protocols. Samples were prepared for DNA fingerprinting by mixing reactions with stop solution (95% formamide, 10 mM NaOH) at a ratio of 2 : 1, heating to 95°C for 3 min, and then immediately snap cooling in an ice bath. A volume of 0.5 *μ*L per lane was spotted onto membrane combs (Gel Company) just prior to the start of electrophoresis. Nondenaturing polyacrylamide gels, 0.5x MDE (Lonza), were mixed according to manufacturer's directions and cast into 61 cm borosilicate plates with 0.2 mm spacers. Fragments were separated on a Licor DNA Analyzer 4300 using 1x TBE running buffer and a gel temperature of 24°C. Gel images were collected as TIF files and opened in ImageJ [20] to generate electropherograms using the freely available ImageJ Gel Analyzer. SSCP electropherograms were converted to a matrix of relative intensity per phylotype to generate a dataset for factor analysis. IBM SPSS Statistics (Windows version) was used to calculate principal components to compare variability of sampling plots L, I, and J over time. 

A number of different terms are used to describe the Archaeal taxa detected with 1D TRFLP-SSCP. *Dominance* is measured as the Archaeal taxon with the highest relative abundance. *Frequency* of detection for a taxon is the number of times it was above the detection limit in a sample. This provides information on spatial distribution both within a plot and within the locality for a given year. *Patchiness* (spatial heterogeneity) also provides information on spatial distribution and is measured as the standard deviation for a taxon listed per year in Table 2. High standard deviations indicate heterogeneous patches exhibiting a wide range of relative abundances, while low standard deviations indicate a more uniform spatial distribution with a similar range of relative abundances. Patchiness can also be visually assessed per plot by inspection of the bar graphs in Figures 5 and 6. 

### 2.3. Clone Libraries and Sequence Analysis

PCR products for cloning were generated as described for 1D TRFLP-SSCP but substituting unlabeled primers. PCR products were then purified with the Promega Wizard SV kit and cloned using the Zero Blunt TOPO PCR Cloning kit (Invitrogen Corp.). Colonies were shipped to the DNA Facility of the Iowa State University Office of Biotechnology for plasmid preparation and Sanger sequencing according to standard protocols. Chromatograms were checked manually for proper base calling, and clones with ambiguous peaks were resequenced so that no ambiguous bases were present in the final contig. Contigs were screened using BLASTN to remove any bacterial sequences and to identify clones that had been isolated in other studies. To remove putative chimeras, sequences were aligned with NAST and then analyzed with Bellerophon at the GreenGenes.lbl.gov website [21]. This resulted in the removal of five chimeric sequences from the dataset. The alignment was checked manually and corrected where necessary to match the greengenes reference alignment and secondary structure predictions. The resulting clone library contained 144 aligned sequences of ~1300 nt. The open source mothur environment [22] was used to calculate operational taxonomic units (OTUs) at various genetic distances using average neighbor clustering. The mothur environment was also used to calculate rarefaction, diversity indices, and AMOVA with Bonferroni correction for repeated measures to compare clone libraries from different years. Simulation studies have shown AMOVA provides an appropriate statistical test to determine whether microbial assemblages are different [23]. To then determine how the clone libraries grouped phylogenetically a maximum likelihood tree was inferred with bootstrap support calculated for each node. Nearest neighbor sequences were chosen, when available, which were at least 1200 nt and within 97% genetic similarity to the cloned sequences. Outgroups at representative taxonomic ranks were added to highlight the position of clades named in the greengenes taxonomy and to illustrate the phylogenetic position of the UA clone WI21. The tree was inferred with PhyML [24] using the GTR model which was selected as the best-fit model for this data set by jModelTest [25] using Akaike information criteria.

### 2.4. Taxonomy

The recently proposed greengenes taxonomy has provided new designations for soil Archaea [26]. To improve readability in this paper, we will use the greengenes prefixes “s_” for species epitaph, “g_” for genus, and “o_” for order, when referring to taxa designated in greengenes. Thus the most frequent and abundant Archaeal taxon in soils, Thaumarchaeota group I.1b [17, 27], corresponds to the newly proposed genus g_*Ca.* Nitrososphaera. Within this genus three taxa have been named at the species level, s_*Ca.* N.* gargensis, *s_SCA1145, and s_SCA1170. The second most frequent and abundant Archaeal taxon in soils, Thaumarchaeota group I.1c, corresponds to a broader taxonomic rank, the order o_NRP-J. Additional taxa within this order have not yet been designated, as no cultivated representatives are currently available. A third Archaeal taxon, Thaumarchaeota group I.1a, is frequently found in marine environments but is also found in low abundance in terrestrial environments. Two I.1a genera that inhabit soil are *Ca. *Nitrosotalea and *Ca.* Nitrosoarchaeum. These have been included in the phylogenetic tree shown in Figure 4. None of the sequences generated in this study grouped in this clade. Finally, Archaeal 16S rRNA gene sequences outside of Thaumarchaeota are persistently found in soil surveys, typically at low abundance [17, 27]. Some of these are methanogens that cluster within Euryarchaeota while others are unclassified Archaeal (UA) sequences distinct from both Euryarchaeota and Thaumarchaeota. 

## 3. Results and Discussion

### 3.1. Comparison of Soil Samples at Stone's Pocket Using SSCP Fingerprinting

To measure persistence of Archaea in soil, we selected a single locality, Stone's Pocket, which contains a common soil, Baraboo silt loam, formed primarily by the deposition of wind-borne dust (Table 1). We first determined variability of soil samples at this locality using Single-Stranded Conformational Polymorphism (SSCP) DNA fingerprinting. 

SSCP DNA fingerprinting characterizes microbial assemblages based on the separation of DNA fragments that differ in sequence composition. Unique sequences have the potential to form a distinct single-stranded secondary structure that will produce a peak (phylotype) when migrating through a polyacrylamide gel. These phylotypes represent one or more unique sequences, and the resulting DNA fingerprint of an assemblage can be used to compare samples in terms of richness (number of phylotypes in the sample) and evenness (the relative abundance of each phylotype). These values can then be converted to a data matrix for principal component analysis (PCA) to visualize the variability in a data set and to conduct statistical testing. 

In 2001, SSCP comparisons of sampling sites A, B, C, G, H, and W (Figure 1(a)) revealed that variability within each site was low enough that three soil samples, each from an initial volume of 3 cc collected along a 30 cm transect, were sufficient to detect statistically significant differences between these locations [18]. 

**Figure 2:**
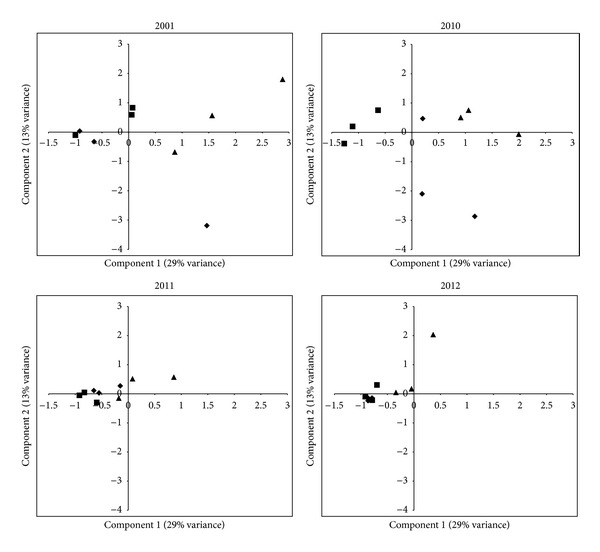
Principal component analysis of SSCP fingerprints from Stone's Pocket. Symbols indicate ▲ plot L, ■ plot I, and *◆* plot J.

At Stone's Pocket we compared the variability of three soil samples collected from plot J, the distant plot, to the variability present in 3 soil samples collected from plots L and I, the adjacent plots (Figure 1(c)). If the diversity of Archaeal assemblages at Stone's Pocket is correlated with distance, the three soil samples from plot J would form a distinct cluster on the PCA ordination plot. On the other hand, if the Archaeal assemblages are not statistically different, then the soil samples from plot J will overlap with L and I. The results show overlap of these three plots at each time point (Figure 2). Soil samples collected from J are located much farther away; plot J is 1.3 km farther; than soil samples from I and L which are within 13 m, yet at each time point sampled, J samples cluster within the variability present in I and L. The PCA ordination plots in Figure 2 demonstrate that our sampling strategy of three plots at Stone's Pocket encompasses the variability detected by SSCP at this locality.

Previous work at site H (Figure 1(a)) used SSCP to sample spatial heterogeneity from three replicate plots within an agricultural field (350 m^2^) revealing an uneven, patchy distribution of phylotypes [18]. In terms of phylotype distribution at Stone's Pocket, a similar uneven distribution was found. Some soil samples from plots L, I, and J contained unique phylotypes that were absent or in low abundance in other samples.

### 3.2. Persistence of Archaeal Assemblages at the Stone's Pocket Collection Site

To investigate how the Archaeal assemblages differ over time at the sequence level, we constructed 16S rRNA gene clone libraries for 2001, 2010, 2011, and 2012. To ensure thatall unique phylotypes were represented in each clone library, five soil samples were pooled per year which together contained all the unique SSCP phylotypes found at each time point. These representative DNA samples included at least one soil sample from each of the three plots at Stone's Pocket. By selecting against soil samples that contained only duplicate phylotypes, the resulting clone libraries are biased to maximize detection of species that fluctuate over time between widespread distribution and limited distribution. 

**Figure 3:**
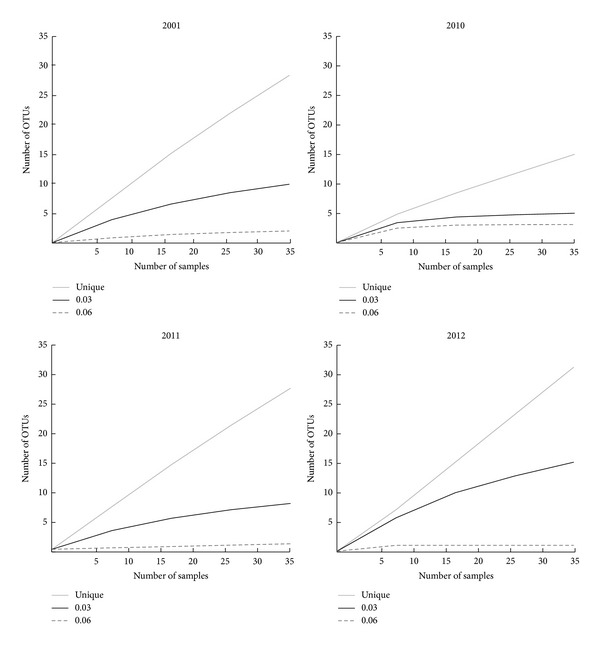
Rarefaction curves of 16S rRNA gene clone libraries from Stone's Pocket. The *y*-axis is number of different OTUs, and the *x*-axis is number of sampled OTUs.

Rarefaction curves for each of the clone libraries indicate high coverage of sequences at some genetic diversity levels (Figure 3). Clone libraries from each year showed a large deflection of the 0.03 genetic diversity curve indicating most of the sequences present in the samples that are within an operational taxonomic unit (OTU) of 97% genetic similarity are included in the libraries. Richness, defined as the number of unique sequences in the clone library, was lowest in 2010 as were alpha diversity indices measured at 0.03 genetic diversity. The Shannon index was 1.6 and Chao was 6 OTUs. To determine whether the Archaeal assemblage changed significantly over time, an AMOVA test was conducted. The clone libraries for each year were found to be unique at a *P* value < 0.01.

**Figure 4:**
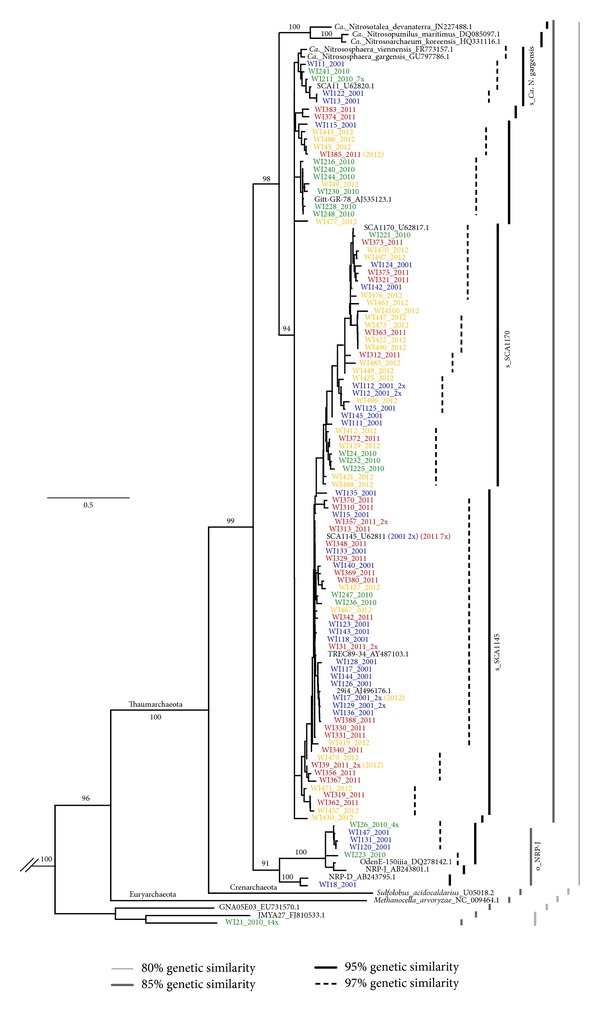
Phylogenetic tree showing the relationships of 16S rRNA sequences recovered from Stone's Pocket over time. Clones recovered multiple times at 100% sequence identity are indicated with the number of times they were identified listed in parentheses. OTUs at various genetic similarities are marked by overlapping bars and are labeled according to the greengenes taxonomy where appropriate. Phyla are labeled at the appropriate branch points; since WI21 is not within an 80% OTU with any known Euryarchaeota, the corresponding branch point is unlabeled. For clarity, only bootstrap values >90% are shown, and some nodes with values <30% were collapsed. The scale bar represents 0.5 changes per nucleotide. A second version of this tree with clone libraries organized by year is depicted in Supplemental Figure 1.

To compare how the libraries are different at each time point, a phylogenetic tree was constructed (Figure 4). This same data is also presented by year for better visualization of the clones at each time point in Supplemental Figure 1 (see Supplemental Figure 1 in supplementary material available online at http://dx.doi.org/10.1155/2013/870825). A number of identical sequences were found multiple times within each year; this is indicated after the clone name in Figure 1, and each duplicate is listed separately in Supplemental Figure 1. To also compare various OTU levels we used average neighbor clustering to place the overlapping bars depicted next to the phylogenetic trees in these figures. Most of the sequences are within the phylum Thaumarchaeota, but in 2010 the most frequently sampled sequence included an unclassified Archaeal (UA) sequence which had less than 80% genetic similarity to Thaumarchaeota and Euryarchaeota. This sequence was below the limit of detection in the other libraries (Supplemental Figure 1). In terms of taxa defined in the greengenes taxonomy, the order o_NRP-J (I.1c) was found in 2001 and again in 2010 but was not detected in the final two sampling years. The species s_SCA1145 and s_SCA1170 were detected each year but varied in abundance while s_*Ca.* N. gargensis was not detected in 2011 and 2012. 

At the level of 100% sequence identity, only one clone matched a sequence previously deposited in Genbank, SCA1145, which was originally cloned 16 times (clones screened by sequencing 400 nt.) from arable soil collected at site W in 1995 [14]. In this study, the SCA1145 sequence (clones screened by sequencing 1300 nt.) was cloned twice in 2001 and seven times in 2011 but was below the limit of detection in the other years. The other sequences that were found at this level in multiple years were WI318 and WI39 which were detected in 2011 and 2012 and WI17 which was detected in 2001 and 2012. 

In summary, the persistence of different taxonomic ranks at Stone's Pocket was found to vary over time. At broad taxonomic ranks, Thaumarchaeota group I.1b was dominant at all time points while o_NRP-J (I.1c) and UA fluctuated from minor constituents of the assemblage to below the detection limit after a time span of one year. In light of these results, we next used multiscale sampling over a wider spatial range to (i) define the niche space that could be dominated by I.1b, I.1c, or UA and (ii) determine over time the stability of these colonized habitats.

### 3.3. Spatial Distribution of Archaeal Assemblages over Time

**Figure 5:**
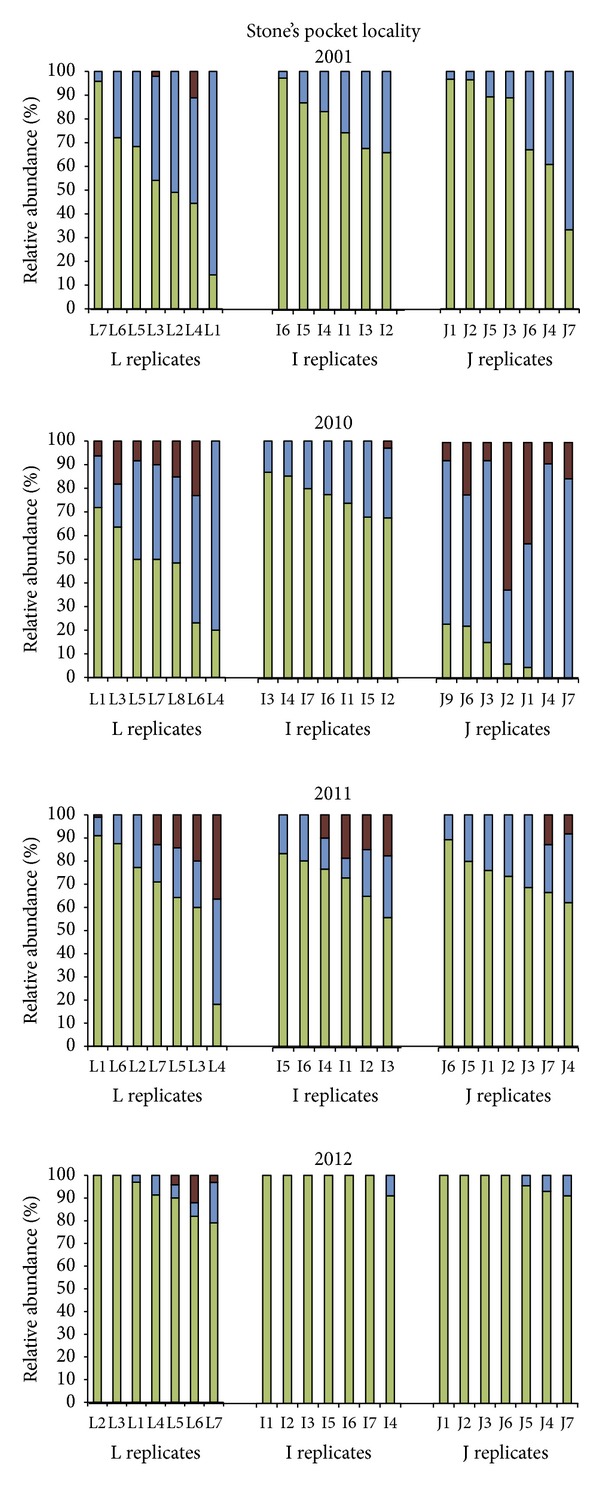
Relative abundance of different taxa at Stone's Pocket measured by 1D TRFLP-SSCP. Stone's Pocket soil samples from plots L, I, and J were grouped according to plot and year. Soil samples within each plot are ordered according to the relative abundance of g_*Ca. *Nitrososphaera (I.1b). Colors represent different taxa: the darkest color, red, indicates unclassified Archaea, the midtone color, blue, indicates o_NRP-J (I.1c), and the lightest color, green, indicates g_*Ca. *Nitrososphaera (I.1b).

**Figure 6:**
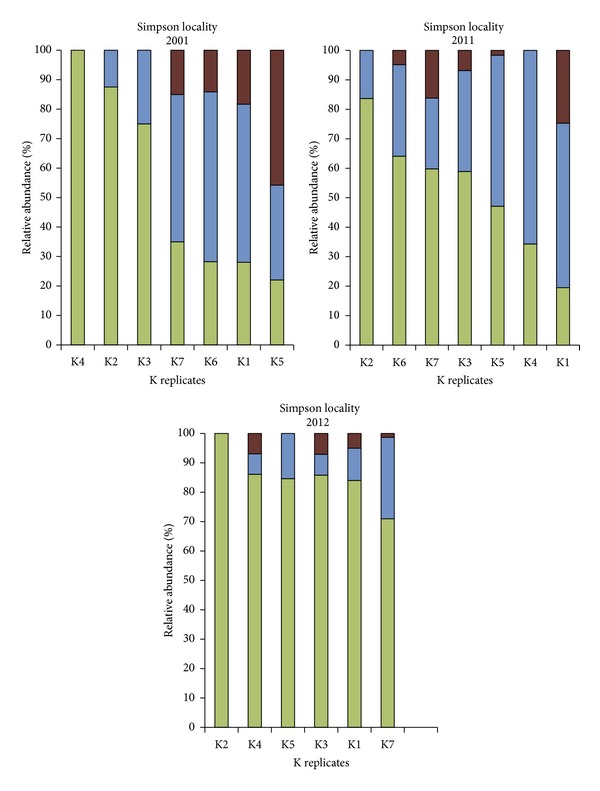
Relative abundance of different taxa at Simpson measured by 1D TRFLP. Soil samples from Simpson plot K are ordered according to relative abundance of g_*Ca.* Nitrososphaera (I.1b). Colors represent different taxa: the darkest color, red, indicates unclassified Archaea, the midtone color, blue, indicates o_NRP-J (I.1c), and the lightest color, green, indicates g_*Ca.* Nitrososphaera (I.1b).

At Stone's Pocket, additional soil samples were profiled using 1D TRFLP-SSCP fingerprinting to investigate spatial distribution of three taxa over time, namely g_*Ca.* Nitrososphaera (I.1b), o_NRP-J (I.1c), and UA. The resulting 1D TRFLP-SSCP electropherograms were converted to the relative abundance graphs grouped by year and by plot in Figure 5. Each bar represents an Archaeal assemblage from a small soil sample, a volume of soil ~3 cubic centimeters (cm^3^). These samples are then organized on increasingly larger spatial scales, first as replicates from the same plot, labeled as L, I, and J replicates. At the next spatial scale, plots L and I are adjacent, and plot J is the distant plot at the Stone's Pocket locality. To sample soil located at a greater distance and at a different locality, plot K at the Simpson collection site was added to this analysis (Figure 1(b)). Simpson is also classified as a temperate mixed forest ecosystem and lies within the same Driftless Area in central Wisconsin but contains a sandier soil that is part of a separate watershed along the Wisconsin River. Collecting samples of Archaeal assemblages at multiple scales allows comparisons to be made over a wider spatial range than a linear sampling strategy. 

Spatial distribution was found to both change and persist over time spans of one year and one decade depending on spatial scale and taxon. At the level of locality, all three taxa were present at the Stone's Pocket and Simpson collection sites, with I.1b consistently dominant at all-time points, followed by I.1c. At the smaller spatial scale of a plot, I.1b dominated all but one plot, I.1c dominated plot J in 2010. Only UA was found to be below the limit of detection at the plot scale. The largest fluctuations occurred at the smallest spatial scale sampled. In these individual soil samples, all three taxa, I.1b, I.1c, and UA, were able to dominate at 100%, 91%, and 63% relative abundance, respectively (Table 2). In addition, it was possible at this spatial scale for each taxa to drop below the detection limit, even the frequently dominant I.1b was absent in some samples, two of the J replicates in 2010. These results indicate that patchiness in heterogeneous environments such as a temperate mixed forest soil occurs at the cm scale, and smaller scales would need to be sampled to find habitats which are dominated solely by o_NRP-J (I.1c) or solely by UA. Additional studies at the microscale level are necessary to define how such patches would form and become inhabited by Archaea.

## 4. Conclusions

This study measured the degree of change for Archaeal taxon composition and patchiness in temperate mixed forest soil. Comparisons of taxa at the sequence level found 2001, 2010, 2011, and 2012, harbored significantly different Archaeal assemblages (AMOVA *P* < 0.01). At the narrow taxonomic ranks that can be detected with 16S rRNA sequences spanning 1300 nt, persistence was found to be rare. Comparing increasingly broader taxonomic ranks showed that Archaea at the species level also fluctuated over time, but the Thaumarchaeota genus g_*Ca.* Nitrososphaera (I.1b) was dominant in all clone libraries. Spatial distribution was characterized at these same time points using 1D TRFLP-SSCP fingerprinting of g_*Ca.* Nitrososphaera (I.1b), o_NRP-J (I.1c), and unclassified Archaea (UA). These taxa formed dynamic patches that fluctuated unevenly over multiple scales of time and distance. The genus g_*Ca.* Nitrososphaera (I.1b) was dominant at most scales and time points, but patches were found within the temperate mixed forest ecosystems that were dominated by o_NRP-J (I.1c) and UA. This study shows that (i) Archaeal assemblage structure changes over time and space and (ii) patch dynamics at the level of cubic centimeters influence Archaeal soil ecology in temperate mixed forest ecosystems. 

## Supplementary Material

Archaeal 16S rRNA sequences recovered from Stone's Pocket listed by year. Each column lists the clones sequenced in that year according to the phylogenetic tree on the left, this allows for visual comparison of the phylogenetic taxa recovered for each year. Clones that were recovered multiple times at 100% sequence identity are listed the number of times they were identified. OTUs at various genetic similarities are marked by overlapping bars and are labeled according to the greengenes taxonomy where appropriate. Phyla are labeled at the appropriate branch points; since WI21 is not within an 80% OTU with any known Euryarchaeota, the corresponding branch point is unlabeled. For clarity, only bootstrap values >90% are shown, and some nodes with values <30% were collapsed. The scale bar represents 0.5 changes per nucleotide.

## Figures and Tables

**Table 1:** Soil classifications.

Label	Sampling site	Soil series	Parent material	Depth to restrictive feature (in)	Drainage class	Depth to water table (in)	Available water capacity	Typical profile			
H^1^	Ag. Station, Hancock	Plainfield sand	Sandy outwash	>80	Excessively drained	~60–80	Low (~3.4 in.)	0–7 in: Sand	7–36 in: Sand	36–70 in: Sand	

A^1^	Ag. Station, Arlington	Plano silt loam	Silty material over sandy loam till	>80	Well drained	~36–60	High (~11.0 in.)	0–13 in: Silt loam	13–45 in: Silty clay loam	45–60 in: Sandy loam	

W^1^	Ag. Station, West Madison	Plano silt loam	Loess over glacial till	>80	Well drained	~42–72	High (~10.7 in.)	0–11 in: Silt loam	11–41 in: Silty clay loam	41–60 in: Sandy loam	

G^1^	Ag. Fields, UW Greenhouses	Colwood silt loam	Stratified silt and fine sand lacustrine deposits	>80	Poorly drained	~0	High (~10.4 in.)	0–10 in: Silt loam	10–24 in: Loam	24–60 in: Stratified fine sand to silt loam	

C^1^	Curtis Prairie, UW Arboretum	Salter sandy loam	Loamy alluvium over stratified silt and fine sand lacustrine deposits	>80	Somewhat poorly drained	~12–36	Moderate (~8.4 in.)	0–10 in: Sandy loam	10–26 in: Loam	26–39 in: Loamy sand	39–60 in: Stratified loamy sand to silt loam

B^1^	Bill's Woods, UW Natural Areas	Kidder loam	Glacial till	>80	Well drained	>80	Moderate (~8.2 in.)	0–9 in: Loam	9–30 in: Sandy clay loam	30–60 in: Sandy loam	

L^2^	Stone's Pocket, Baraboo Hills	Baraboo silt loam	Loess over quartzite	20–40	Moderately well drained	~24–36	Low (~5.7 in.)	0–5 in: Silt loam	5–34 in: Silty clay loam	34–38 in: Unweathered lithic bedrock	

I^2^	Stone's Pocket, Baraboo Hills	"	"	"	"	"	"	"	"	"	

J^2^	Stone's Pocket, Baraboo Hills	"	"	"	"	"	"	"	"	"	

K^2^	Simpson, Baraboo Hills	Eleva sandy loam	Loamy residuum weathered from sandstone	20–40	Well drained	>80	Low (~4.0 in.)	0–9 in: Sandy loam	9–27 in: Sandy loam	27–36 in: Sand	36–60 in: Weathered paralithic bedrock

^1^Sites sampled previously [22].

^2^Plots sampled over multiple years in this paper.

Ag.: agricultural.

UW: University of Wisconsin, Madison, WI, USA.

**Table 2:** Comparison of relative abundance of soil Archaea over time and between localities.

locality		g_*Ca.* Nitrososphaera				o_NRP-J				Unclassified Archaea			
	Year	Average (%)	Max	Min	Stdev	Average (%)	Max	Min	Stdev	Average (%)	Max	Min	Stdev
Stone's Pocket	2001	70	97	14	23	29	86	3	22	1	11	0	3
	2010	45	87	0	31	43	91	13	25	12	63	0	16
	2011	67	89	18	16	23	45	8	9	9	36	0	11
	2012	94	100	79	7	5	19	0	6	<1	4	0	1

Simpson	2001	54	100	22	33	33	58	0	22	13	46	0	16
	2011	52	84	19	21	40	66	16	18	8	25	0	9
	2012	85	100	71	9	13	28	0	9	2	7	0	3
