# Supplementary figures and images for: Archaeal Assemblages Inhabiting Temperate Mixed Forest Soil Fluctuate in Taxon Composition and Spatial Distribution over Time
Source: Archaea. 2013 Aug 1;2013:870825. doi: 10.1155/2013/870825 (PMC3747363; doi:10.1155/2013/870825)

**2001 2010 2011 2012**

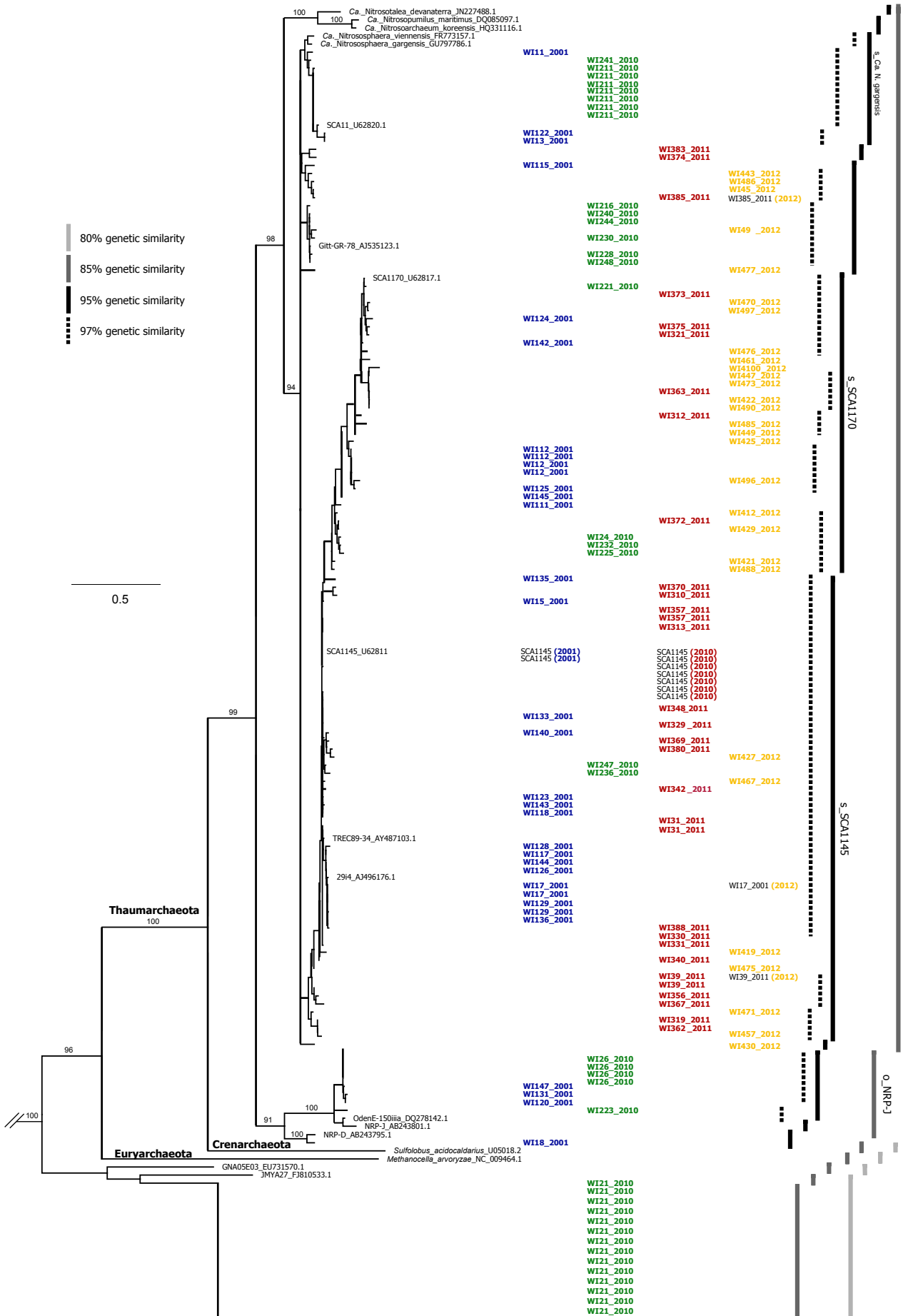

Supplement: Supplementary file 1 — Archaeal 16S rRNA sequences recovered from Stone's Pocket listed by year. Each column lists the clones sequenced in that year according to the phylogenetic tree on the left, this allows for visual comparison of the phylogenetic taxa recovered for each year. Clones that were recovered multiple times at 100% sequence identity are listed the number of times they were identified. OTUs at various genetic similarities are marked by overlapping bars and are labeled according to the greengenes taxonomy where appropriate. Phyla are labeled at the appropriate branch points; since WI21 is not within an 80% OTU with any known Euryarchaeota, the corresponding branch point is unlabeled. For clarity, only bootstrap values >90% are shown, and some nodes with values <30% were collapsed. The scale bar represents 0.5 changes per nucleotide. [file 870825.f1.pdf]
